# Effects of Sensory Garments on Sleep of Children with Autism Spectrum Disorder

**DOI:** 10.1155/2022/2941655

**Published:** 2022-02-12

**Authors:** Lisa Mische Lawson, Lauren Foster, Morgan Hodges, Mia Murphy, Melissa O'Neal, Lesan Peters

**Affiliations:** Department of Occupational Therapy Education, University of Kansas Medical Center, USA

## Abstract

**Objective:**

The purpose of this study is to assess the effectiveness of the use of sensory garments for improving sleep in children with autism spectrum disorder.

**Method:**

Using a single-subject ABAB reversal design, the researchers evaluated the effectiveness of a sensory garment on sleep duration, sleep latency, and parental stress related to a child's sleep. Four children aged 4–10 participated. We measured sleep duration and sleep latency using the Garmin watches and parent-report sleep logs, parent stress using the Parenting Stress Index Short Form, and sleep behaviors using the Children's Sleep Habits Questionnaire. *Results/Discussion*. Data showed variable effects on sleep duration and latency across children. The oldest child with the hyposensitive sensory patterns experienced the greatest sleep improvements. All parents experienced stress from daily life, and some reported increased stress due to study participation. Future research is recommended to further investigate the effectiveness of sensory garments on sleep for children with ASD. Therapists are encouraged to evaluate children's development and sensory preferences prior to recommending sensory garments for sleep.

## 1. Introduction

Autism spectrum disorder (ASD) is a neurodevelopmental disorder that impacts 1 in 54 children [1]. The core diagnostic features include “persistent deficits in social communication and interaction” and “restrictive, repetitive patterns of behavior, interests or activities” [2]. However, ASD is a spectrum, meaning that the disorder presents differently in each individual and uniquely affects their lives. Additionally, 86% of children with ASD often experience at least one comorbid condition including the intellectual disabilities, attention deficit and hyperactivity disorder, gastrointestinal symptoms, enuresis, epilepsy, and sleep disturbances [3, 4].

Children with ASD are also more likely to experience sensory modulation disorders compared to typically developing children [5], with up to ninety-five percent of children with ASD exhibiting sensory processing dysfunction [6, 7]. According to Dunn's [8, 9] model of sensory processing, children with sensory differences recognize and respond to sensory stimuli differently than their peers and may have hyperactive or hypoactive responses to the stimuli. An individual's neurological threshold is the level at which one notices and responds to sensory stimuli. Children with low sensory thresholds frequently notice stimuli (hypersensitive) when others do not, whereas children with high sensory thresholds require more sensory stimuli (hyposensitive) to register sensory input. Children with sensory differences may also react strongly to stimuli (hyperreactive) or take a passive approach (hyporeactive). Emerging evidence suggests that sensory processing patterns are linked to sleep disturbances, particularly hypersensitive responses to touch [10].

Sensory-based interventions target problem behaviors by helping the person modulate sensory processing needs through environmental modifications (e.g., low lighting) and/or physical modification (e.g., weighted vests) [11]. Occupational therapists often recommend sensory-based interventions to help improve sensory modulation in children with ASD [12]. However, despite their prevalence, evidence regarding the effectiveness of sensory-based interventions such as weighted vests, massage, and therapy balls is mixed [11, 13].

Research suggests that 40–80% of children with ASD also experience increased sleep disturbances, including insomnia, night waking, delayed onset of rapid eye movement sleep due to an increase in the time falling asleep, and an overall decrease of total sleep time especially in comparison to their typically developing peers [5, 14]. Sleep challenges children with ASD experience may be influenced by how they process and respond to sensory stimuli [5, 10]. Specifically, hypersensitivity and sensory dysregulation are known to be associated with insomnia for a subset of children with ASD [15]. Additionally, differences in sensory processing are reported to have significant associations with sleep difficulties for children with ASD [16].

Children's sleep troubles have also shown to have emotional, economic, social, and educational implications on the family unit [17]. Nearly half of all families of children with ASD experience increased stress related to ASD comorbidities, such as sleep issues [18]. Parents may also experience lower sleep totals and quality of sleep because of their child's sleep troubles and a lower sense of competence in their parenting [17]. Additionally, research suggests that children's sleep difficulties lead to higher maternal depression rates and marital discord which may contribute to higher divorce rates.

Occupational therapists recognize sleep as an essential occupation across the lifespan. Sleep quality influences quality of life, health, and meaningful engagement in occupations [19]. Sleep can also impact attention, executive functioning, and overall cognitive performance [20, 21]. Common occupational therapy interventions to support sleep include developing consistent bedtime habits and routines, cognitive-behavioral therapy, physical modifications to the environment, and sensory-specific strategies [22]. For example, seminal autism research of deep pressure techniques for children with ASD utilized anecdotal evidence which demonstrated inconsistent effectiveness [23]. Recently, weighted blankets have become a popular and easily accessible sensory tool. However, there is no evidence to suggest weighted blankets improve sleep in children with ASD [24]. Compression garments, which provide equal distribution of pressure and help regulate the sensory system, are an emerging, wearable product that provides deep pressure input [25]. However, evidence to support these products is limited; thus, more research is needed to determine their effectiveness. The purpose of this study was to investigate the effects of a compression t-shirt on the sleep of children with ASD. Secondarily, we investigated if changes in children's sleep impacted parent stress.

## 2. Methods

### 2.1. Design

This study utilized an ABAB single-subject design to examine the effects of sleep with a small sample of children. The single-subject design allows children to serve as their own control when evaluating outcomes before and after an intervention has been provided. This is beneficial due to the individualized characteristics present in people with ASD [26].

### 2.2. Participants

This study utilized convenience sampling to recruit participants. Participant recruitment occurred through closed Facebook groups, departmental email lists, word of mouth, and community organizations. Eligible participants met the following inclusion criteria: (i) a diagnosis of autism spectrum disorder (ASD), (ii) four to twelve years of age, (iii) located in the Kansas City area, (iv) parent indication of child sleep difficulties, (v) a score of 56 or greater on the Social Responsiveness Scale, and (vi) at least one sensory pattern outside of the typical range indicated on the Child Sensory Profile 2. Researchers provided participants with a $100 incentive after the completion of all study activities.

### 2.3. Measures

The screening measures included a demographics questionnaire, the Child Sensory Profile 2 (CSP2; [27]), and the Social Responsiveness Scale, 2nd edition (SRS-2; [28]). The demographics questionnaire provided information on participant race/ethnicity, sex, age, height, weight, diagnoses, and current medications and services related to ASD. The CSP2 [27] is a caregiver questionnaire that measures sensory processing in children aged 3–14 years, and the SRS-2 [28] is a caregiver report questionnaire that measures the severity of ASD in children.

The assessment measures included the Children's Sleep Habits Questionnaire (CSHQ; [29]), the Parenting Stress Index-Short Form (PSI-SF; [30]), and the Garmin Forerunner 735XT or 935. The CSHQ is a caregiver report measure that includes the child's bedtime and night walking behavior, sleep onset and duration, anxiety, sleep-disordered breathing, and daytime sleepiness. Five times during the study, parents reported their child's sleep habits and difficulties related to sleep during a typical week using the CSHQ. The PSI-SF is a parental self-report measure that indicates responses to life stressors. Parents reported changes in the weekly stress level using the PSI-SF. Participants used the Garmin Forerunner 735XT or 935 watches nightly throughout the seven weeks to gather the specific sleep metrics of the heart rate, light sleep, deep sleep, and sleep duration [31]. The Garmin was chosen over other objective sleep measures (e.g., Bedditt), as the best option for continually measuring sleep of children who may leave their bed. Researchers encouraged participants to wear the watch two hours prior to bedtime per Garmin's wearing recommendations for accurate sleep data collection [31]. For participants who did not tolerate the Garmin, a parent-reported sleep log collected sleep latency and sleep duration data. The researchers conducted parent interviews after the conclusion of data collection to gain insight on parent perceptions of the compression garment and their child's sleep.

### 2.4. Design and Procedures

The University of Kansas Medical Center Human Subjects Committee (#00146392) approved this study. Participants provided informed consent after eligibility screening prior to beginning study activities. Child participants were encouraged to provide informed assent when deemed able by their parents. Interested families completed the demographics form, the CSP-2, and the SRS to determine eligibility. A Garmin Forerunner 735XT or 935 watch collected sleep-related data for one week during the first baseline phase (A1). Next, during the first intervention phase (B1), participants wore the Garmin watches and a SmartKnitKids Compresso-T for three weeks. Then, participants removed the Compresso-T for a second week of a baseline phase (A2). To end, participants wore the Compresso-T for a second intervention phase (B2) of two weeks. Caregivers completed the CSHQ and PSI-SF at each phase including before week one, after week one, after week four, after week five, and after week seven.

### 2.5. Analysis

Single-subject design research looks at outcomes before and after the intervention while allowing focus on one individual's outcomes related to specific diagnoses [26]. Researchers collected nightly Garmin or sleep log data to be graphed and analyzed for consistency of response and rate of behavior change. Specific sleep data analyzed were night awakenings, sleep duration, and sleep onset. Researchers calculated mean SRS-2 and CSHQ summary scores.

## 3. Results

Four children with atypical sensory patterns (Table 1) aged 4–10 with ASD participated in the study. All participants completed all study activities; however, two children were unable to tolerate wearing the Garmin watch and used parent-reported sleep logs as an alternative. All children wore the Compresso-T with modifications (e.g., cutting larger neck holes) or other strategies (e.g., wearing under pajamas) to encourage wear. During the data collection process, some instances of missing or incomplete data occurred due to intolerance of the Garmin watch, vacations, shift in parental responsibilities, and/or technical difficulties. Additionally, daylight savings time began during the first intervention period, and the study occurred during the winter months of the COVID-19 pandemic (February–April 2020).

### 3.1. Child 1

Child 1 was a 4-year-old Caucasian male diagnosed with ASD. He was 44 inches tall and weighed 44.5 pounds. He received occupational therapy, sleep-language therapy, and applied behavioral analysis services. He took NovaFerrum, an iron supplement, once daily and began taking ½ or 1 mg of guanfacine daily during week three of the study. His SRS-2 score of 97 indicated severe difficulties in the reciprocal social behavior that leads to severe interference with everyday social interactions. His bedtime was between 7:30 pm and 8:00 pm, and his typical wake time was between 7:00 am and 8:00 am. He occasionally took naps at school or fell asleep on the bus. His bedtime routine consisted of a bath, applying lotion and oils, and reading a book. He used two weighted blankets when sleeping that are less than five pounds each. He frequently moved to his parents' bed during the middle of the night and slept there until he woke up. He tolerated the Garmin and Compresso-T for the duration of the study. Due to technical issues with the Garmin, his parents began recording a sleep log as well.

**Figure 1:**
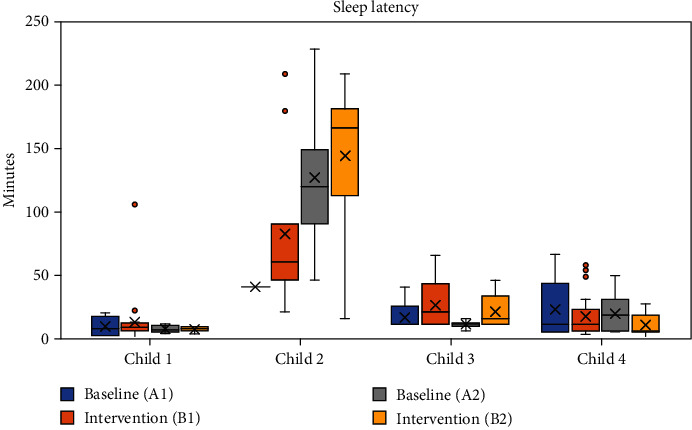
Sleep latency. *Note*. Child 2 and 3 latency is the time to fall asleep reported by parents. Child 1 and 4 latency is the time from light to deep sleep reported by the Garmin. Child 1 has 12 days of missing latency data. Only one latency data point was available for Child 2 during A1 with a total of 17 days of missing latency data. Child 3 has 6 days of missing latency data. Child 4 has 2 days of missing latency data. For bar graph analysis, missing data was excluded.

**Figure 2:**
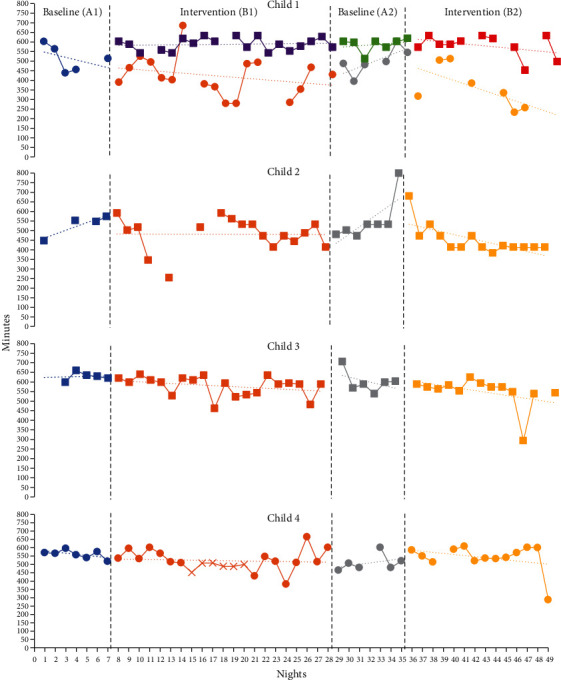
Sleep duration. *Note*. Garmin data is identified by a circle. Sleep log data is identified by a square. Child 4 spring break is marked by an X during intervention (B1). We did not input missing data.

Sleep latency decreased steadily throughout the study with the first baseline (A1) being the longest and the second intervention (B2) being the shortest (Figure 1). Sleep duration appeared to worsen during the intervention phases (B1, B2) when compared to the baseline phases (Figure 2). It is important to note the differences in sleep duration between the parent-reported sleep log and the Garmin watch that the child wore when sleeping (Figure 2). The total scores from the CSHQ showed lower scores during the intervention phases versus the baseline phases. However, the difference in scores between phases is minimal (Tables 2 and 3). The parent reported,

I felt like I saw a change that first week we had it [compression garment] on. He still might have woken up but he definitely seemed to sleep deeper per the watch at least. As time went on I do not know if maybe his body desensitized to it.

PSI-SF scores indicated slightly less stress during the intervention phases compared to the baseline phases. There is also a consistent downward trend across all phases (Table 2). The parent reported,

I was more stressed about if he would wear it [the compression garment] or not. I think in the beginning I was concerned about if he would wear it or if I would mess up the study for you. Once I was getting used to doing it every night, I did not feel the stress of worrying about it.

Though the Compresso-T did not appear to improve sleep latency or duration consistently or meaningfully, the parent reported sleep improvements not captured by study measures:

He does this really super fun thing where he gets euphoric sometimes before bedtime, really wound up, really excited, instead of getting relaxed. And so I think when he was wearing the garment, we had less of those nights. I would say that it probably did make him relax and decreased the euphoric stuff.

### 3.2. Child 2

Child 2 was a 4-year-old Caucasian male with ASD. His SRS-2 score of 87 indicates severe difficulties with reciprocal social behavior. He was 36 inches tall and weighed 37 pounds. He received occupational therapy, speech-language therapy, and special education services. He took 2 mg of guanfacine twice daily and 9 mg of melatonin 30 minutes before bed for the duration of the study. His parents reported no bedtime routine and that his typical sleep cycle is between 7:00 pm and 5:00 am where he stays in his bed for the entire night. He had no history of sensory garment use for sleep. He tolerated the watch for a few weeks and then transitioned to a parent report sleep log. The family travelled for one week of vacation during the second baseline (B2) phase.

Throughout the study phases, mean sleep latency increased. The longest instance of sleep latency occurred during the second baseline (A2), and shortest instance occurred during the second intervention phase (B2) (Figure 1). Only one data point was available the first baseline period (A1) due to difficulties tolerating the watch before switching to a parent directed sleep log. During both baseline phases (A1 and A2), an upward trend line revealed increased sleep duration. Intervention (B1) showed a consistent trend line throughout the phase with minimal change, while the trend line for intervention (B2) sloped downward towards decreased sleep duration (Figure 2). It is important to note that the parent reported that “he liked wearing it … [and] he responds well to deep pressure type input like weighted blankets and compression … they help him regulate.” The CSHQ revealed the greatest amount of sleep difficulty prior to the beginning of the study. After both intervention phases (B1, B2), sleep difficulties slightly increased (Tables 2 and 3). The parent reported that the use of the garment “seemed to only help [his sleep] for a week … but like everything else we've tried, it had a very short shelf life.” The PSI-SF scores indicated the greatest amount of parent stress prior to the beginning of the study (Table 2). The scores decreased after baseline (A1) and remained consistent throughout the duration of the study (Table 2). Additionally, the parent reported that her “stress levels stayed pretty high all of the time … the things with his needs that cause me stress have not significantly changed [including] sleep, behavioral issues, and scheduling doctors' appointments.”

### 3.3. Child 3

Child 3 was a 4-year-old Caucasian female with ASD. Her SRS-2 score of 88 indicated severe difficulties with reciprocal social behavior. She attended school at a local preschool and received occupational therapy, physical therapy, and speech therapy services. She took no medications. Her parents reported a bedtime routine including putting on pajamas, brushing her teeth, reading books in bed, turning off the lights, and her mother lying in bed with her until she fell asleep. Her typical sleep cycle was from 8:15 pm to 6:30 am. Most nights she moved to her parents' bed and slept the remainder of the night with them. She occasionally took 45- to 90-minute naps during the day. She had a history of wearing a stabilizing pressure input orthosis (SPIO) suit to school and previously used a weighted blanket during sleep. For this study, her parents made modifications to the watchband, but she did not tolerate the Garmin watch, so parents utilized a parent-reported sleep log to collect data. Her parents stated that the Compresso-T “was tough to get over her head so she didn't like it. We just snipped it in the middle at the chest on the neckline”. Her parents reported that she tolerated and did not resist the Compresso-T once on her body.

For Child 3, the sleep latency mean was shorter in the baseline phases and longer in the intervention phases (Figure 1). Mean sleep latency was longest during the first intervention phase (B2) and shortest during the second baseline phase (A2) (Figure 1). Longer sleep duration occurred during the first and second baseline phases (A1 and A2) (Figure 1). However, the downward sloped trend lines for the first intervention (B1), second baseline (A2), and second intervention (B2) denote decreased sleep from the beginning to the end of the phase (Figure 2). The CSHQ revealed the least amount of sleep difficulty prior to the beginning of the study. Minimal changes occurred in sleep difficulties from prior to the beginning of the study to the end of the first baseline, except in sleep resistance, which improved during the intervention periods (Tables 2 and 3). The parent reported, “I just don't think it made a difference what she wore” regarding the parent's perception of if sleep improved during the study. PSI scores indicated the least amount of parent stress prior to the beginning of the study (Table 2). Scores greatly increased from prior to the beginning of the study to after baseline (A1) (Table 2). Stress decreased slightly after the first intervention phase (A1), but overall remained consistently high throughout the study (Table 2). Additionally, the parent reported, “I'm not going to lie I had anxiety … every night knowing I had to put it on her it was a little stressful but once it was on it was fine.”

### 3.4. Child 4

Child 4 was a 10-year-old Caucasian male with ASD. His SRS-2 score of 144 indicated severe difficulty with reciprocal social behavior. He was 57 inches tall and weighed 90 pounds. He received special education services and was homeschooled. He took a multivitamin, magnesium, and Vyvanse the duration of the study. His parents reported a bedtime routine of a nightly bath and that his typical sleep cycle is between 9:00 pm and 8:00 am where he stayed in his bed for the entire night. He used a weighted blanket nightly for sleep. Screen time was rewarded before bed 2–3 days weekly based on behavior. He tolerated the watch with no issues and after given an incentive he tolerated the Compresso-T for the duration of the study. He engaged in a week-long vacation for spring break during the first intervention phase (B1) and was sick for one of the nights.

For Child 4, mean sleep latency was shorter in the intervention phases when compared to the baseline phases and the mother stated, “I don't feel like it has been taking him as long to go to sleep.” (Figure 1). Sleep duration recorded with the Garmin was consistent throughout the intervention, though the mother stated, “He was sleeping longer the longer he wore the garment” (Figure 2). Parent-reported sleep issues increased during the first baseline period and then remained the same throughout the duration of the study (Tables 2 and 3). The CSHQ revealed that sleep onset delay worsened, but sleep latency decreased according to Garmin data and parent report (Tables 2 and 3). Parent Stress Index scores indicated that the parent had lower stress before the study began and experienced higher stress scores during the intervention phases; the mother stated, “I do not feel like I had any extra added stress from the study (Table 2).”

## 4. Discussion

Children responded variably to the garment based on sensory preferences and age. The oldest child with the hyposensitive sensory patterns experienced the most changes in sleep patterns from the intervention. Child 4 was the only child who fell into the “much more than others - low registration” category of the Sensory Profile 2. Children with this pattern are hyposensitive to sensory input and tend to miss cues in their environment [27]. Theoretically, these children will benefit from pressure garments because pressure garments increase physical input during the task. While we must be cautious in generalizing these results, the case provides an argument for the need to examine each child's sensory processing patterns before recommending sensory-based interventions.

Research shows factors contributing to sleep problems in children with ASD differ with age [32]. Older children and adolescents report more problems with sleep onset, suggesting the shorter sleep latency or quicker sleep onset that Child 4 experienced is meaningful given his age. It is possible younger children did not show improvements in sleep latency because research indicates younger children tend to fall asleep faster than older children. Though children in this study showed variable response to the intervention, the three younger children all had shorter sleep latency at baseline than Child 4. In addition to differences in sleep onset, Goldman et al. [33] found that younger children display more resistance behaviors during bedtime, compared with older children and adolescents. Thus, the improvements in bedtime resistance demonstrated by Child 3 suggest that specific sleep outcomes may be important to consider rather than total sleep scores, or the sleep outcomes most reported (e.g., sleep latency and sleep duration).

Age may have also influenced the children's ability to fully comply and benefit from study participation. Children develop the ability to self-regulate as they age (e.g., with the significant changes occurring in the first 5–7 years) (e.g., [34]). By around age 7, children have developed active self-regulation strategies and require fewer external supports to self-regulate [35]. Since self-regulation and sleep are related (e.g., [36, 37]) and self-regulation continues to develop throughout childhood, older children should have better skills at regulating sleep. Also, children do not develop abstract reasoning and flexible problem-solving skills until around six years of age [38]. Therefore, the older child may have been able to understand the purpose of the compression garment compared to younger children and better comply with wearing it to produce sleep improvements.

Results of this study are consistent with previous research that suggests children with ASD respond variably to sleep interventions, with older children with specific sensory preferences more likely to benefit [39]. Additionally, the Garmin only captured consistent data from the oldest child. Though Child 1 consistently wore the Garmin, he frequently woke for long periods of time at night and often moved to his parents' bed. The Garmin registered the child as awake “for the day” after the child was active for several minutes and would no longer record sleep data even when he fell back to sleep for several hours. It is possible parent-reported sleep logs were not sensitive enough to capture improvements resulting from the sensory garment [40, 41]. Also, sleep logs may not capture some important sleep outcomes for young children, such as bedtime resistance, parasomnias, or anxiety.

Previous literature has found a negative association between ASD severity and sleep duration [42], suggesting children with more severe ASD have greater sleep issues and get less sleep. In our study, all children had “severe difficulties with reciprocal social behavior” based on their SRS-2 scores. Contrary to previous research, in our study, Child 4 had the greatest severity of autism symptoms but did not have the greatest sleep issues. He was also the only child who displayed a uniformly hyporesponsive sensory processing pattern, indicating a preference for more sensory input. Though he did not have the greatest sleep issues, he was the only child to experience consistent, meaningful improvement because of the garment suggesting age and sensory preferences may be as or more important than autism severity to benefit from some interventions. Additionally, research has shown that some individuals with ASD have difficulties with sensory regulation and may lack the ability to habituate to sensory stimuli [43] particularly, touch and sound. A pressure garment, although potentially helpful for a child who is hyporesponsive to proprioceptive input, may not decrease the stress response of a child who is sensitive to touch and sound. However, parents believed their child habituated to the deep pressure sensation provided by the compression garment. This suggests that compression garments may not be an effective long-term solution for sleep.

We hypothesized that improving sleep would decrease parent stress. This study found variable and minimal sleep improvements, so it is unlikely sleep was associated with parent stress for this study. Yet, all parents in this study demonstrated increased levels of stress during the interventions phases or reported consistently high levels of stress throughout the intervention. Parents reported stress related to engaging in the study, particularly with encouraging the child to wear the garment. It is possible that research participation impacted parent stress more than changes in child sleep patterns [44]. It is also possible parents' increased stress impacted children's sleep over the course of the study. Evidence suggests behavioral sleep education may be helpful for reducing parent stress and increasing parent competence for supporting children's sleep [45]. Combining behavioral sleep education with sensory strategies, like sensory garments, may be more effective for improving sleep of children with ASD. Most research examines interventions focused on improving child sleep to reduce parent stress [14]; however, it may be helpful to address parent stress to improve child sleep.

## 5. Strengths and Limitations

Specific strengths within this study include maintaining children's daily routines and schedules and observing sleep in the child's natural context. The single-subject design allowed for individualized data collection and analysis. This study included both objective and parent-reported data regarding parent stress, sleep duration, and sleep latency. Single-subject research designs allow for flexibility and manipulation of interventions specific to each participant while still maintaining consistent procedures. Additionally, all participants completed the study indicating both the study intervention and design were feasible with this population.

Some children had difficulties tolerating wearing the Garmin watch and technical difficulties created disruptions in data collection for multiple families. Participants in this study were encouraged to continue daily life activities and did not control for changes in medications. While this allowed for naturalistic investigation, regular life events potentially impacted results. Lastly, we recruited a volunteer sample that may have been more motivated than typical families to improve their children's sleep.

## 6. Future Research

Considerations for future research include comparing the differences in sleep quality using compression garments between younger and older children, assessing effectiveness of garments on children with specific sensory patterns (e.g., hyposensitive), and exploring alternative options for objectively measuring sleep, such as actigraphy. Researchers should consider combining sensory garments along with behavioral sleep education to best address sleep of children with ASD. Subsequent studies should also seek to understand how stress is related to children's sleep, particularly by exploring how reducing parent stress and increasing parent competence impacts sleep. Additionally, future research should consider measuring sleep outcomes based on age to best capture meaningful change.

## 7. Implications for Practice

Current literature on the effectiveness of deep pressure techniques and garments for sleep varies. This study adds to this variable research. Therapists should utilize discretion and consider the child's and family's individualized needs, age, and sensory processing patterns when suggesting compression garments to children to improve sleep. Parent stress *increased* in three of the four participants, suggesting therapists should (i) use caution when recommending a sensory garment and (ii) consider contextual and family factors when implementing a sensory-based sleep intervention. Additionally, it is important to note that compression garments are not intended to be used as independent interventions [46] and might be best combined with behavioral education strategies to improve sleep hygiene [47]. Therapists should also consider how a child's sleeping habits and patterns may influence parental stress, or alternately how parent stress may influence child sleep, when working with children and families. Finally, because reliance on routines is a hallmark of ASD [2], therapists should use caution when recommending any changes to the bedtime routine without a full evaluation of the child's developmental history and sensory processing patterns.

## 8. Conclusion

Comorbidities including sleep difficulties are prevalent in children with ASD. Compression garments are an emerging wearable product to support sleep; however, current evidence to support deep pressure compression garments is limited. This single-subject study contributes to the body of evidence regarding compression garment use for children with ASD. This study found varied and inconsistent effects of compression garments on sleep duration, sleep latency, and parental stress. Additional studies are needed to determine if sensory garments are an effective sleep aid and explore child characteristics related to best response.

## Figures and Tables

**Table 1:** Sensory preferences.

	Child 1	Child 2	Child 3	Child 4
Seeking/seeker	=	=	++	=
Avoiding/avoider	+	+	++	=
Sensitivity/sensor	++	=	++	=
Registration/bystander	++	++	++	++
Auditory	+	=	+	=
Visual	+	=	++	=
Touch	++	+	++	+
Movement	++	=	++	++
Body position	++	++	++	++
Oral	+	=	++	=
Conduct	=	=	++	=
Social emotional	+	+	++	=
Attentional	+	=	++	=

^∗^ *Note*. Much less than others = --. Less than others = -. Just like the majority of others = =. More than others = +. Much more than others = ++.

**Table 2:** Parent Stress Index and Children's Sleep Habit Questionnaire total scores for each child.

		Testing period				
		1^∗^	2	3	4	5
Child 1	PSI total	83	98	96	88	83
	CSHQ total	111	108	106	111	107
Child 2	PSI total	136	82	82	79	84
	CSHQ total	122	114	122	109	118
Child 3	PSI total	81	156	136	162	164
	CSHQ total	110	118	117	117	117
Child 4	PSI total	120	137	140	132	137
	CSHQ total	110	115	115	115	115

^∗^ *Note.* 1: before baseline; 2: after baseline. before introducing intervention; 3: after 1^st^ intervention phase; 4: after 2^nd^ baseline phase; 5: after 2^nd^ intervention phase.

**Table 3:** Children's Sleep Habit Questionnaire total scores and subscale scores for each child.

		Testing period				
		1^∗^	2	3	4	5
Child 1	Total	111	108	106	111	107
	Bedtime resistance	21	20	21	20	19
	Sleep onset delay	3	4	2	2	2
	Sleep duration	8	8	8	8	8
Child 2	Total	122	114	122	109	118
	Bedtime resistance	19	24	24	24	25
	Sleep onset delay	4	3	3	4	4
	Sleep duration	9	6	8	4	5
Child 3	Total	110	118	117	117	117
	Bedtime resistance	21	19	17	19	17
	Sleep onset delay	2	4	2	3	2
	Sleep duration	6	10	10	9	9
Child 4	Total	110	115	115	115	115
	Bedtime resistance	24	24	24	24	24
	Sleep onset delay	2	1	5	5	5
	Sleep duration	6	6	6	6	6

^∗^ *Note*. 1: before baseline; 2: after baseline, before introducing intervention; 3: after 1^st^ intervention phase; 4: after 2^nd^ baseline phase; 5: after 2^nd^ intervention phase.

## Data Availability

Contact the corresponding author for data.
